# Q Fever Knowledge, Attitudes and Vaccination Status of Australia’s Veterinary Workforce in 2014

**DOI:** 10.1371/journal.pone.0146819

**Published:** 2016-01-12

**Authors:** Emily Sellens, Jacqueline M. Norris, Navneet K. Dhand, Jane Heller, Lynne Hayes, Heather F. Gidding, Harold Willaby, Nicholas Wood, Katrina L. Bosward

**Affiliations:** 1 Faculty of Veterinary Science, The University of Sydney, Sydney, NSW, Australia, 2006; 2 School of Animal and Veterinary Sciences, Charles Sturt University, Locked Bag 588, Wagga Wagga, NSW, Australia, 2678; 3 National Centre for Immunisation Research and Surveillance, Locked Bag 4001, Westmead, NSW, Australia, 2145; 4 School of Public Health and Community Medicine, UNSW Medicine, University of New South Wales, Sydney, NSW, Australia, 2052; 5 Sydney School of Public Health, The University of Sydney, NSW, Sydney, Australia, 2006; 6 Discipline of Paediatrics and Child Health, Sydney Medical School, The University of Sydney, Sydney, NSW, Australia, 2006; University of Minnesota, UNITED STATES

## Abstract

Q fever, caused by *Coxiella burnetii*, is a serious zoonotic disease in humans with a worldwide distribution. Many species of animals are capable of transmitting *C*. *burnetii*, and consequently all veterinary workers are at risk for this disease. An effective Q fever vaccine has been readily available and used in Australia for many years in at-risk groups, and the European Centre for Disease Prevention and Control has recently also called for the use of this vaccine among at-risk groups in Europe. Little is known about attitudes towards this vaccine and vaccine uptake in veterinary workers. This study aimed to determine the Q fever vaccination status of veterinarians and veterinary nurses in Australia and to assess and compare the knowledge and attitudes towards Q fever disease and vaccination of each cohort. An online cross-sectional survey performed in 2014 targeted all veterinarians and veterinary nurses in Australia. Responses from 890 veterinarians and 852 veterinary nurses were obtained. Binary, ordinal and multinomial logistic regression were used to make comparisons between the two cohorts. The results showed that 74% of veterinarians had sought vaccination compared to only 29% of veterinary nurses. Barriers to vaccination among those not vaccinated did not differ between cohorts, and included a lack of perceived risk, financial expense, time constraints, and difficulty in finding a vaccine provider. Poor knowledge and awareness of Q fever disease and vaccination were additional and notable barriers for the veterinary nursing cohort, suggesting veterinary clinics and veterinarians may not be meeting their legal responsibility to educate staff about risks and risk prevention. Further evaluation is needed to identify the drivers behind seeking and recommending vaccination so that recommendations can be made to improve vaccine uptake.

## Introduction

Q fever is a serious zoonotic disease capable of causing chronic debilitating and life threatening, illness in humans [1]. Following infection by the causative bacterium *Coxiella burnetii*, about 40% of patients become symptomatic in the acute phase, with symptoms most often limited to a flu-like illness. However, 2–5% of acute Q fever patients develop severe complications such as hepatitis, atypical pneumonia, myocarditis or meningitis [1–5]. Acute infection during pregnancy has been associated with miscarriage, foetal death, premature delivery and low birth weights, with women in their first trimester at greatest risk [6–8].

Chronic Q fever may develop months or years after acute infection, with immunocompromised or pregnant patients predisposed, as are those with pre-existing heart valve lesions, vascular disease or prosthetic joints [8,9]. Endocarditis is the most common presentation of chronic Q fever reported [9,10]. Chronic Q fever fatigue syndrome (QFS) is another well recognised sequelae to acute Q fever, occurring in up to 10% of patients, and is related to the persistence of *C*. *burnetii* antigens within the host in the absence of viable bacteria [11–13]. Women with chronic Q fever may experience recurrent miscarriage or pre-term deliveries [7]. Due to the non-specific and variable presentations of both acute and chronic Q fever, diagnosis may be delayed in the absence of a high index of suspicion, prolonging illness and endangering the lives of those affected [14,15].

*C*. *burnetii* is shed in greatest numbers in the products of conception, and to a lesser extent the urine, faeces, milk and saliva of infected animals [1,16]. The bacterium exhibits a spore-like stage of its lifecycle, which is extremely resistant to environmental and chemical insults allowing for survival outside the host for longer than 12 months [1,17,18]. Inhalation is the most common route of infection, with *C*. *burnetii* easily dispersed by wind over large areas [19].

A wide variety of domestic and wild animal species act as a reservoir for *C*. *burnetii*. To date most human infections have been attributed to cattle, sheep and goats [1] which has led to a general belief that Q fever is a disease of production animal workers and those living in rural communities. However, studies in Australia and Japan have demonstrated seroprevalence for *C*. *burnetii* to be similar among people in rural and metropolitan areas of some regions [20,21] suggesting that alternative sources such as companion and wild animals may be an under-recognised contributor to Q fever infection [22]. Indeed there are an increasing number of reported cases of Q fever in humans that have been attributed to companion animals [23–26]. Parturient cats have been implicated across the globe since the 1980’s [23] with cats now considered a significant source of Q fever disease in Japan and Maritime Canada [3,23] and a potential source of disease in Korea, the United States of America (USA), the United Kingdom and Australia [26–31]. Dogs and horses have also been identified as a source of Q fever disease around the world [24,25,32–34]. With a broad range of species capable of transmitting *C*. *burnetii*, the occupational risk to veterinary workers is undeniable, regardless of their practice type. Outbreaks among veterinary workers have been associated with direct or indirect contact with birth products following cat and dog caesareans in small animal veterinary clinics [25,26,35]. Studies in Japan, Denmark, the Netherlands and the USA have confirmed a significantly higher seropositivity to *C*. *burnetii* among veterinarians compared to the general population [21,36–38], while Q fever was the second most common zoonosis reported among Australian veterinarians in a recent survey [39].

A whole cell formalin-inactivated Q fever vaccine (Q-Vax®; CSL Biotherapies, Parkville, Vic.) has been available in Australia since 1989 and has a reported efficacy of over 98% [40]. To date, routine use and licensing has been restricted to Australia, in part due to a perception that the vaccine is “old-fashioned” and concerns regarding adverse events following immunisation [41,42]. Any persons who have previously had Q fever or exposure to *C*. *burnetii* should not receive the vaccination due to an increased risk of adverse events following immunisation [43]. Strict pre-vaccination protocols have been successfully implemented in Australia to minimise the risk of adverse events; serology and intradermal skin testing with diluted vaccine to check for evidence of prior exposure. This process requires experienced medical practitioners and may be seen as costly and time consuming [44].

Currently, the Australian Veterinary Association (AVA) Biosecurity Guidelines and the Australian Immunisation Handbook recommend vaccination of all veterinarians, veterinary students and veterinary nurses [45,46]. The vaccination process is now a course requirement for students enrolled in veterinary and animal science degrees at Australian universities. Outside of this tertiary environment, Q fever vaccination may be recommended or a compulsory requirement to commence employment for some veterinary and other animal workers.

The European Centre for Disease Prevention and Control has released recommendations for the use of this vaccine in targeted groups, including veterinary workers, in European countries while a new-generation vaccine is being developed [41]. The implementation of routine Q fever vaccination internationally would come at some expense, and lessons from the Australian experience could help to mitigate cost and time.

The aim of this study was to determine the Q fever vaccination status and compare the knowledge, attitudes and practices of veterinarians and veterinary nurses in Australia to Q fever with the further aim of informing vaccine policy both in Australia and internationally and making recommendations to maximise workplace health and safety (WH&S) for all veterinary personnel.

## Methods

### Study Design

This cross-sectional study was targeted at all veterinarians and veterinary nurses in Australia over 18 years of age and currently or recently employed in a veterinary workplace. The study was implemented via the Survey Monkey® (Palo Alto, California, USA) platform as an online questionnaire containing 53 questions (13 open, 25 closed and 15 semi-closed) divided across six sections; (1) demographics and veterinary work environment, (2) attitudes towards Q fever illness and vaccination, (3) experience with Q fever disease, (4) experience with Q fever vaccination, (5) knowledge of disease risk, and (6) biosecurity practices. Skip logic was used and it was not compulsory to answer all questions. Ethics approval was granted by Charles Sturt University School of Animal and Veterinary Sciences Human Ethics Committee (protocol #416/2013/19). A participant information statement was provided to participants and informed consent was sought prior to commencement of the survey.

### Recruitment of veterinary nurses

Veterinary nurses were recruited during March and April of 2014. Due to limitations in accessing this unique workforce, which requires no formal registration outside of the state of Western Australia, participants were recruited in a number of ways. A personal email invitation containing a link to the survey was sent on our behalf by the Veterinary Surgeon’s Board of Western Australia to all veterinary nurses in this state using the email address listed with the board. In other states and territories, attempts were made to phone all veterinary clinics to invite veterinary nurses to participate in the survey. The clinic phone lists for New South Wales and Tasmania were compiled from practice lists provided by the state’s respective veterinary practitioner’s boards whilst the remaining state lists were compiled from all clinics listed with the Yellow Pages^®^ phone book. Reminder emails/letters/faxes were sent out two weeks after the first email, letter or fax. The Veterinary Nursing Council of Australia (VNCA) also sent personal emails to members for whom they had an email address recorded.

### Recruitment of veterinarians

Initial contact with veterinarians was made through an invitation advertised in the AVA’s email newsletter sent to all members on the 11^th^ of April 2014. In May and June of 2014 veterinarians were recruited in a similar fashion to veterinary nurses. Personal email invitations were sent on our behalf by state veterinary surgeons boards where possible. In other states, the contact lists compiled for distributing the survey to clinics for participation of veterinary nurses were revised for the recruitment of veterinarians. Clinics that had previously declined participation of their veterinary nurses were contacted to invite participation from veterinarians and phone calls were also made to check method of contact preference where post or fax had been previously specified. Reminder emails/letters/faxes were sent out 2 weeks after the first. During May 2014, the Veterinary Practitioners Board of NSW also provided details and a link to the survey on their website, which had increased traffic during this month as it coincided with veterinarians’ registration renewals.

### Data management and analysis

Binary, ordinal and multinomial logistic regression analyses were undertaken to compare the knowledge, attitudes and practices between veterinarian and veterinary nurse cohorts (the exposure) and all models were adjusted for age, sex and state to account for demographic differences of the cohorts. P-values of <0.05 were considered statistically significant and 95% confidence intervals (CI) were calculated. For ordinal outcomes, the assumption of proportionality was evaluated using the Score Test. Where the Score Test was found to be significant (p<0.05), indicating that the assumption was invalid, categories were combined to create binary variables or if appropriate multinomial logistic regression was undertaken.

Agreement with attitudinal statements regarding the importance of, potential harm from, and difficulty in accessing the Q fever vaccination were compared between the two cohorts using binary logistic regression with the positive outcome ‘agree’. Q fever knowledge was assessed as self-reported knowledge, with participants asked to rate their level of Q fever knowledge on a scale of one (lowest) to ten (highest), and a Kruskal-Wallis Test undertaken to assess for a statistical difference between the mean rank of each cohort. Perception of vaccine safety, efficacy and expense was compared with ordinal logistic regression. The positive outcome “agree/strongly agree” was modelled over lower levels of agreement in veterinarians compared to veterinary nurses. Self-perceived level of risk (nil, low, moderate, high) of personal exposure to *C*. *burnetii* was compared between the two cohorts using multinomial logistic regression. Odds ratios were calculated for veterinarians versus veterinary nurses, with logits modelled using ‘nil’ exposure as the reference category. Respondents were considered to have attempted vaccination if they reported that they had been vaccinated or were positive at pre-vaccination screening. Odds of attempting vaccination and odds of receiving vaccination were compared using separate binary logistic regression models with the positive outcomes ‘attempted vaccination’ and ‘vaccinated’, respectively. Multinomial logistic regression was used to determine the odds of vaccination of each cohort across the three most likely vaccination scenarios; (1) actively sought vaccination despite no workplace or study requirement to do so, (2) vaccinated as a requirement of work (3) vaccinated due to a university/ other course requirement.

Practice structures were defined as ‘solo’ (one veterinarian within the clinic), ‘group’ (multiple veterinarians within the clinic), ‘corporate’ (multiple veterinarians with a clinic owned and managed by a corporate entity), ‘university’ (clinical, research and/or academia within a university) or ‘other’. Practice type by species was determined by the combination of species with which respondents spent >90% of their working hours with. Responses stating “don’t know” or “unsure” were excluded from all comparisons. All analyses were performed in SAS® statistical program (2002–2012 SAS Institute Inc; Cary, NC, USA).

## Results

### Sampling

A total sample size of 1,742 participants was achieved, comprised of 852 veterinary nurses and 890 veterinarians.

Of the 995 veterinary nurses registered with the Western Australia state veterinary board, 113 were not contactable for this survey due to the absence of a registered email address. The remaining 882 were individually emailed an invitation to participate, although 74 of these emails were subsequently undeliverable. In other states of Australia, phone calls were made to 1,677 clinics inviting participation of veterinary nurses. Of the 1,446 clinics who agreed to receive participation details, 1,286 preferred to receive an email link to the survey (54 of these emails were subsequently undeliverable), while 91 were sent survey details via post and 69 via fax. Personal emails were sent to 917 VNCA members.

Sampling of veterinarians conducted via email invitations sent on our behalf by state veterinary practitioners boards resulted in individual emails sent to 1200 veterinarians in Western Australia and 245 veterinarians in Tasmania. The number of veterinarians registered with these state boards and the number of undeliverable email invitations were not able to be obtained. In the remaining states contacted initially via phone, 1582 clinics agreed to participate with 1458 sent an email link to the survey (23 of these emails were undeliverable), while survey details were sent to 82 clinics via post and 42 clinics via fax. It is not known how many veterinarians accessed the survey via the AVA’s email newsletter (which was viewed by 2537 members), or the NSW veterinary surgeon’s board website.

### Demographics and veterinary work

Although an accurate response rate was unable to be determined from this survey due to the absence of a single registry by which all veterinarians and veterinary nurses could be contacted, it was estimated that participation by 890 veterinarians and 852 veterinary nurses represented approximately 12% of the estimated 7,400 employed veterinarians and 10% of the estimated 8,600 employed veterinary nurses at the time according to Australian government employment statistics [47,48]. All states and territories were represented The majority of veterinary nurses were female (98%), compared to 63% of veterinarians, and the nursing cohort was, both in range of years and on average, younger and reported fewer years in the veterinary workforce (Table 1). Both cohorts mostly worked in small animal practice with a group practice structure predominating (Table 1). The reported number of staff in clinics ranged from one to 299 with a median number of ten and interquartile range of ten, indicating that most clinics would be categorised as small businesses. In terms of education, most (65%) veterinary nurses had completed Certificate IV level training at a technical tertiary institution although 13% reported no formal education. The veterinarians responding to the survey were mostly graduates of Australian universities (89%), with all seven Australian veterinary schools represented, and 35% of respondents held additional postgraduate qualifications (S1 Table).

**Table 1:** Demographics and veterinary work of participants in study of Q fever knowledge attitudes and practices in 2014 in Australia.

	Veterinarians (n = 890)	Veterinary Nurses (n = 852)
Characteristic	No.(%)^a^	No.(%)^a^
**Sex**		
Female	560 (63%)	836 (98%)
Male	321 (36%)	14 (2%)
Not specified	9 (1%)	2 (<1%)
**Age**		
Range	21–80 years	18–69 years
Mean	40 years	33 years
Median	38 years	31 years
Standard deviation	12 years	10 years
Interquartile Range	19 years	16 years
18–30 years	251 (28%)	403 (48%)
31–40 years	238 (27%)	229 (27%)
41–50 years	202 (23%)	144 (17%)
51+ years	194 (22%)	68 (8%)
**Years working**		
Range	0.2–60 years	0.3–47 years
Mean	16.2 years	10 years
Median	14 years	8 years
Standard deviation	12 years	8 years
Interquartile Range	19 years	10 years
0.2–5 years	192 (22%)	305 (37%)
6–10 years	172 (20%)	222 (27%)
11–20 years	225 (26%)	219 (26%)
21–30 years	157 (18%)	64 (8%)
31+ years	128 (15%)	21 (3%)
**Practice Type**		
Small animals	512 (58%)	640 (75%)
Farm/mixed animals	297 (33%)	132 (15%)
Equine/other	37 (4%)	17 (2%)
Not specified	44 (5%)	63 (7%)
**Practice Structure**		
Corporate^b^	32 (4%)	48 (6%)
Group^c^	575 (65%)	441 (52%)
Solo^d^	169 (19%)	256 (30%)
University	31 (3%)	36 (4%)
Other	45 (5%)	19 (2%)
Not specified	38 (4%)	52 (6%)

^a^Unless specified otherwise. Percentages are of total respondents for each parameter. Not all participants responded to all questions.

^b^One veterinarian within the clinic

^c^Multiple veterinarians within the clinic

^d^Multiple veterinarians within a clinic owned and managed by a corporate entity.

### Attitudes towards vaccination

The majority of both cohorts (97%) agreed that vaccines in general are important in the prevention of disease. In comparison with veterinary nurses, veterinarians had higher odds (2.18; 95% CI 1.44–3.38; p<0.001) of being convinced of the importance of the Q fever vaccine and lower odds (0.53; 95% CI 0.38–0.74; p<0.001) of being concerned that the vaccine may be harmful (Table 2). Greater than 40% of each cohort agreed that the vaccine was difficult to access, with veterinarians reporting lower odds (0.77; 95% CI 0.60–0.97; p = 0.03) of agreement with this statement (Table 2).

**Table 2:** Binary logistic regression analysis of attitudes towards the Q fever vaccine among veterinarians and veterinary nurses surveyed in Australia in 2014.

	Agree	Disagree	Total	Adjusted Odds Ratio^a^^b^	95% CI^c^	P-value^d^
***"If a vaccine exists for a certain disease*, *then vaccination is usually a good way to protect someone against this disease"***						
Nurses	727 (97%)	20 (3%)	747	1		
Vets	805 (97%)	22 (3%)	827	1.13	0.55–2.35	0.75
***"I am convinced of the importance of the Q fever vaccine"***						
Nurses	645 (88%)	86 (12%)	731	1		
Vets	765 (93%)	54 (7%)	819	2.18	1.44–3.38	<0.001
***"I worry that the Q fever vaccine will do more harm than good"***						
Nurses	131 (18%)	600 (82%)	731	1		
Vets	96 (12%)	723 (88%)	819	0.53	0.38–0.74	<0.001
***"It is difficult to get vaccinated for Q fever"***						
Nurses	312 (44%)	404 (56%)	716	1		
Vets	327 (41%)	479 (59%)	806	0.77	0.60–0.97	0.03

^a^Odds of stating “agree”

^b^Ratio adjusted for age, sex and state

^c^Confidence interval

^d^Likelihood ratio Chi-square p-value.

### Knowledge and perceptions of Q fever vaccination and disease

**Fig 1:**
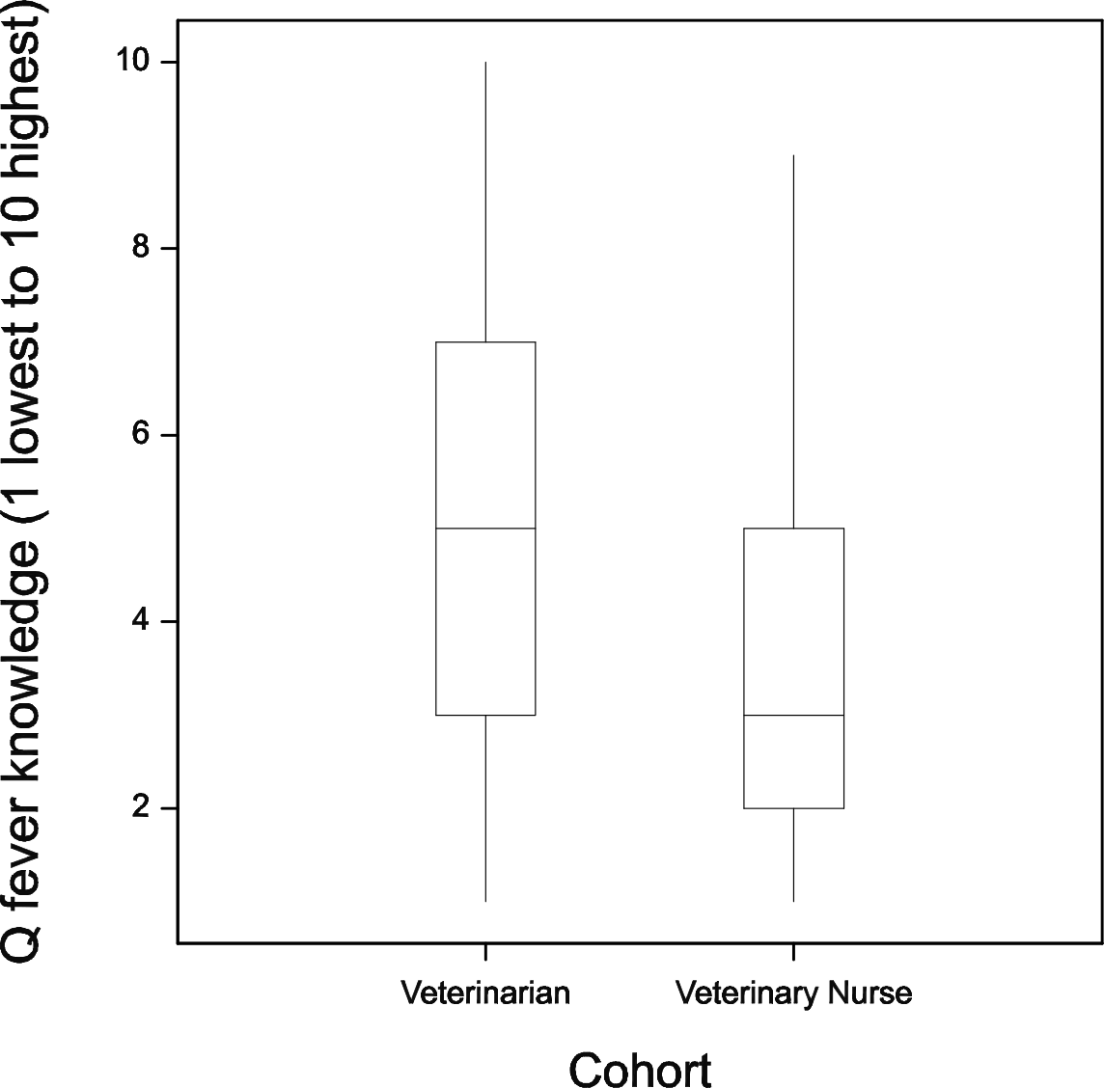
Boxplot of self-rated Q fever knowledge among veterinarians and veterinary nurses.

The majority of both cohorts (98%) agreed (slightly agreed/agreed/strongly agreed) that Q fever is a serious disease with significant health consequences. Self-reported Q fever knowledge on a scale of one (lowest) to ten (highest) was normally distributed among veterinarians (mean = 5; median = 5), while that of veterinary nurses was positively skewed (mean = 3.5; median = 3) (Fig 1). The Kruskal-Wallis Test identified a significant difference (p<0.001) in knowledge between the cohorts. Veterinarians had 1.5 times (95% CI 1.1–2.1; p = 0.03) odds of agreeing that the vaccine is safe, 3.3 times (95% CI 2.2–5.1; p<0.001) odds of agreeing that the vaccine is effective and 0.35 times (95% CI 0.26–0.47; p<0.001) odds of agreeing that the vaccine is expensive compared to veterinary nurses (Table 3). The cost, safety and efficacy of the Q fever vaccine was not known among 43% (342/802), 13% (102/807) and 21% (168/805) of veterinarians and 50% (358/716), 26% (187/722) and 31% (221/722) of veterinary nurses respectively.

**Table 3:** Ordinal logistic regression analysis of the perceptions of the Q fever vaccination among veterinarians and veterinary nurses surveyed in Australia in 2014.

	Strongly disagree/ disagree	Slightly disagree	Slightly agree	Agree/ strongly agree	Total	Adjusted OR^a^^b^	95% CI	P-value^c^
***"The Q fever vaccine is safe if appropriately administered"***								
Nurses	4 (<1%)	12 (2%)	78 (15%)	441 (82%)	535	1		
Vets	3 (<1%)	19 (3%)	71 (10%)	612 (87%)	705	1.49	1.05–2.13	0.027
***"The Q fever vaccine is effective in preventing Q fever"***								
Nurses	5 (1%)	15 (3%)	84 (17%)	397 (79%)	501	1		
Vets	1 (<1%)	6 (1%)	43 (7%)	587 (92%)	637	3.3	2.19–5.08	<0.001
***"The Q fever vaccine is too expensive"***								
Nurses	90 (25%)	50 (14%)	100 (28%)	118 (33%)	358	1		
Vets	236 (51%)	71 (15%)	76 (16%)	78 (17%)	461	0.35	0.26–0.47	<0.001

^a^Odds Ratio: odds of stating “agree/strongly agree” modelled over the lower levels of agreement. Assumption of proportionality met.

^b^Adjusted for age, sex and state.

^c^Wald Chi-square P-value.

### Exposure to *C*. *burnetii*

**Fig 2:**
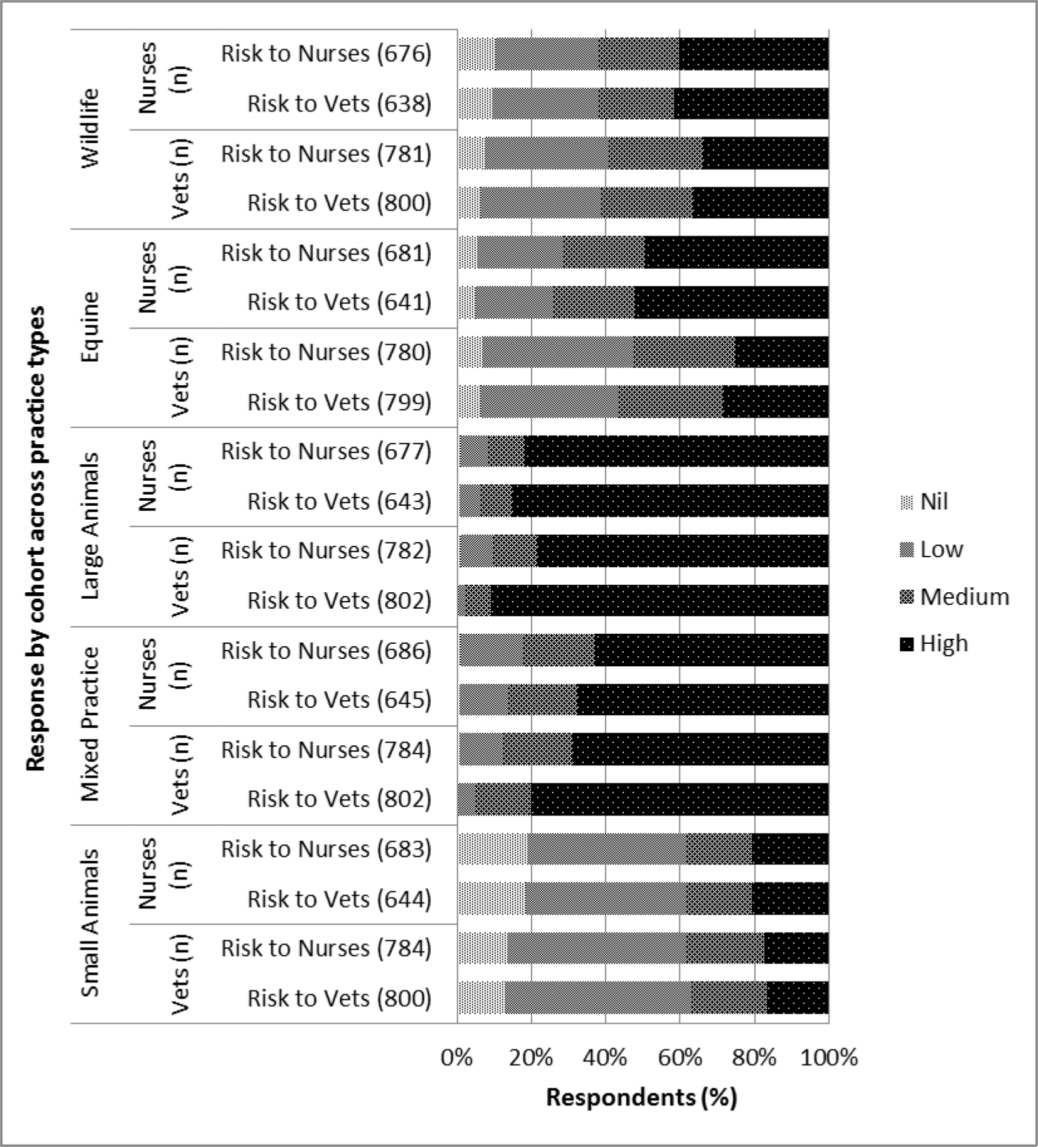
Perceived exposure risk of veterinarians and veterinary nurses to *C*. *burnetii* across different practice types.

Both cohorts considered the risk of exposure to *C*. *burnetii* to be similar for both veterinarians and veterinary nurses within each practice type (Fig 2). Ordinal regression analysis of perceived level of exposure to *C*. *burnetii* did not meet the assumption of proportionality and binary categories were not appropriate. Subsequently multinomial regression was undertaken which, when adjusted for practice type, revealed veterinarians had 12.5 times (95% CI 6.4–25.1; p<0.001) the odds of stating their personal risk as high rather than nil compared to veterinary nurses (Table 4). Alarmingly, 11% (93/850) of veterinarians and 25% (201/793) of veterinary nurses stated they “did not know” their level of exposure and a further 11% (150/793) of veterinary nurses stated ‘nil exposure’. Of those veterinary nurses stating ‘nil exposure’, 91% worked within small animal practices, 7% in large and mixed animal practices, and the remainder in equine or other practices.

**Table 4:** Multinomial logistic regression analysis of level of perceived personal exposure to *C*. *burnetii* among veterinarians versus veterinary nurses surveyed in Australia in 2014.

	Nil	Low Exposure				Moderate Exposure				High Exposure			
	n (%)	n (%)	Adjusted OR^a^	95% CI	p-value^b^	n (%)	Adjusted OR^a^	95% CI	p-value^b^	n (%)	Adjusted OR^a^	95% CI	p-value^b^
**Nurses (n = 793)**	151 (19%)	329 (41%)	1			88 (11%)	1			24 (3%)	1		
**Vets (n = 850)**	33 (4%)	414 (49%)	5.4	3.5–8.7	<0.001	216 (25%)	8.2	4.8–13.9	<0.001	94 (11%)	12.5	6.4–25.1	<0.001

^a^Odds Ratio; logits modelled using "nil exposure" as the reference category and adjusted for age, sex, state and practice type.

^b^Wald Chi-square p-value.

### Vaccination status and barriers to vaccination

The majority of veterinarians (587/796; 74%) were either vaccinated (488/587; 61%) or had sought vaccination for Q fever but were unable to be vaccinated due to a positive pre-vaccination screening result (99/587; 12%). This proportion increased to 78% (562/721) among graduates of Australian veterinary schools and decreased to only a third (25/75; 33%) among international graduates (Table 5). Only 29% (199/688) of veterinary nurses had been vaccinated (162/199; 24%) or had sought vaccination but were unable to be vaccinated due to a positive pre-vaccination screening result (37/162; 5%) (Table 5). Overall, veterinarians had 13 times (95% CI 9.9–18.1; p<0.001) odds of having attempted vaccination and 10 times (95% CI 7.6–12.6; p<0.001) odds of having received the vaccination.

**Table 5:** Q fever vaccination status of veterinarians and veterinary nurses surveyed in Australia in 2014.

	All vets (n = 796)	Vets graduated in Australia (n = 721)	Vets graduated internationally (n = 75)	Nurses (n = 688)
	*n (%)*	*n (%)*	*n (%)*	*n (%)*
***Attempted vaccination***				
Vaccinated	488 (61%)	478 (66%)	10 (13%)	162 (24%)
Pre-screen positive	99 (12%)	84 (12%)	15 (20%)	37 (5%)
Total attempted	587 (74%)	562 (78%)	25 (33%)	199 (29%)
***Not attempted vaccination***				
Not aware of the vaccine	57 (7%)	38 (5%)	19 (25%)	205 (30%)
Aware of the vaccine	152 (19%)	121 (17%)	31 (41%)	284 (41%)
Total not attempted	209 (26%)	159 (22%)	50 (67%)	489 (71%)

Among those who had attempted vaccination, a positive pre-vaccination screening result was reported by 17% (99/587) of veterinarians and 19% (37/199) of veterinary nurses. Among veterinarians graduating from Australian veterinary schools this percentage fell slightly to 15% (84/562) while 60% (15/25) of international graduates who had attempted vaccination were found to be positive at pre-vaccination screening.

Veterinarians mostly (81%) received their vaccination as a requirement of a university course while veterinary nurses were commonly vaccinated as a job requirement (43%). Multinomial regression revealed veterinary nurses had twice the odds (95% CI 1.2–5.0; p = 0.02) of having been vaccinated as a job requirement than having actively sought vaccination outside of a job requirement than veterinarians, while veterinarians had 19 times (95% CI 9.1–40.7; p<0.001) odds of having been vaccinated as a university/course requirement than having actively sought vaccination outside of a job requirement than veterinary nurses.

Among respondents who had not attempted Q fever vaccination, 27% (57/209) of veterinarians and 42% (205/489) of veterinary nurses were not aware the vaccine existed (Table 5). Reasons for non-vaccination among those aware of its existence did not differ significantly between the two cohorts, with the perception that “I will not be seriously affected by Q fever” identified as the most influential reason for not seeking vaccination (Table 6).

**Table 6:** Proportional odds of the influence of known barriers to vaccination among veterinary nurses versus veterinarians surveyed in Australia in 2014.

	Influence			Total	Adjusted OR^a^^b^	95% CI	p-value^b^
	Nil n(%)	Minor/ Moderate n(%)	Major/ sole n(%)				
***Unable to access a Q fever vaccine provider***							
Nurses	194 (71%)	39 (14%)	41 (15%)	274	1		
Vets	98 (66%)	28 (19%)	22 (15%)	148	0.66	0.34–1.28	0.21
***Q fever vaccine may not be effective***							
Nurses	234 (86%)	35 (13%)	4 (1%)	273	1		
Vets	128 (87%)	17 (12%)	2 (1%)	147	1.65	0.65–4.62	0.305
***Unable to afford the financial cost of vaccination***							
Nurses	173 (63%)	65 (24%)	36 (13%)	274	1		
Vets	116 (78%)	22 (15%)	10 (7%)	148	1.19	0.60–2.43	0.628
***Q fever vaccination may be harmful***							
Nurses	211 (77%)	55 (20%)	8 (3%)	274	1		
Vets	109 (74%)	34 (23%)	4 (3%)	147	0.91	0.45–1.91	0.648
***Pre-screening and vaccination process is too time consuming***							
Nurses	198 (72%)	61 (22%)	15 (5%)	274	1		
Vets	100 (68%)	31 (21%)	15 (10%)	146	0.53	0.26–1.06	0.063
***Perception that "I won't be seriously affected by Q fever"***							
Nurses	134 (49%)	82 (30%)	60 (22%)	276	1		
Vets	72 (49%)	35 (24%)	41 (28%)	148	1.04	0.58–1.88	0.893

^a^Odds Ratio: odds of stating “major/sole influence” modelled over the lower levels of influence. Proportionality assumption was met.

^b^Wald Chi-square p-value.

Seven participants who were not vaccinated commented that their medical practitioner had little knowledge of, or had advised against, Q fever vaccination. Such comments included;

*“I have seen two GP's…regarding Q fever vaccination… neither one had any real idea of what was involved… one looked it up and made me feel the risks of vaccination were too high*.*”*

*“My doctor when questioned about the existence of this vaccine did not believe that it existed*.*”*

*“My doctor was under the impression you only need this vaccine if you are travelling overseas*.*”*

### Sources of biosecurity information

**Fig 3:**
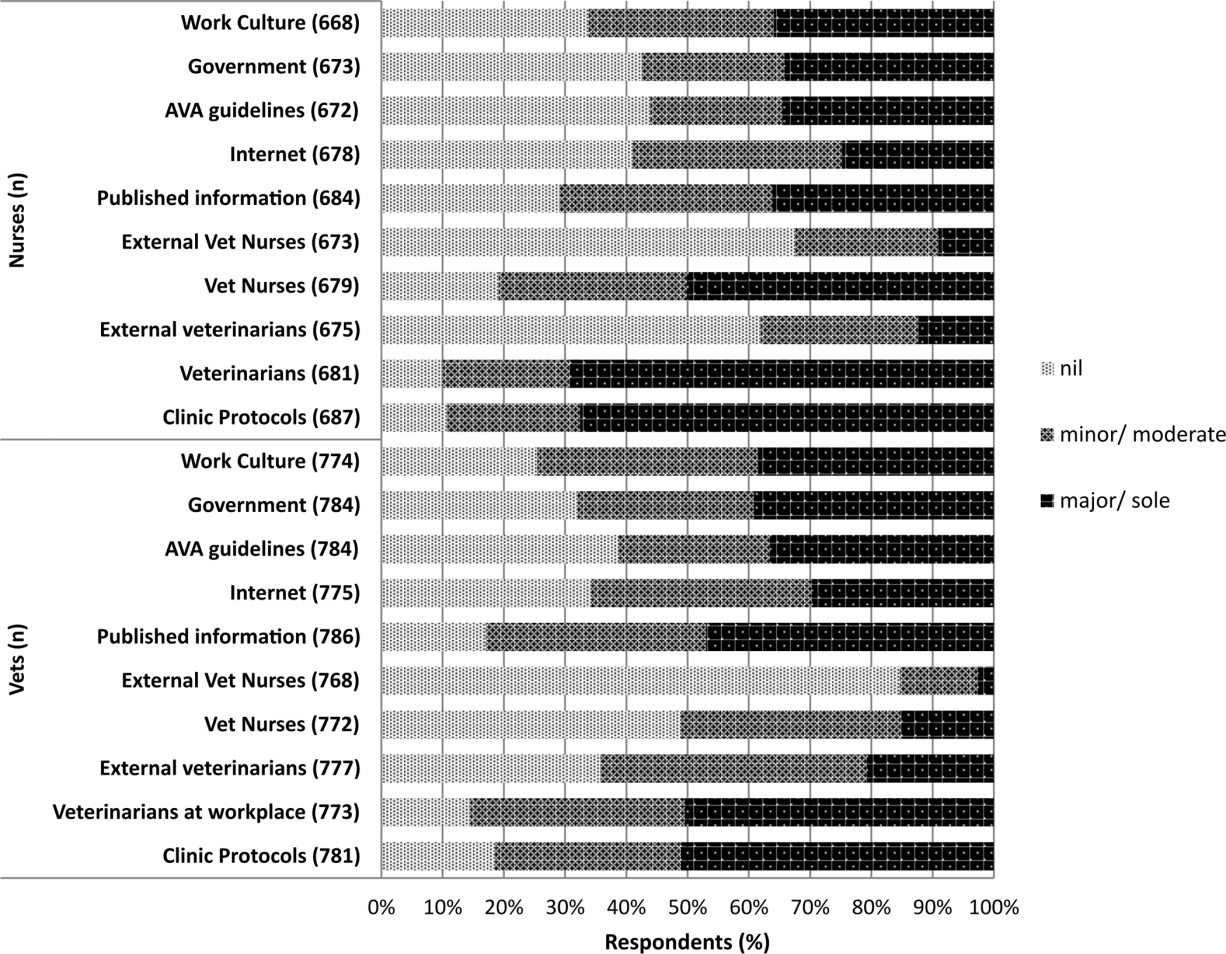
Level of influence of sources of biosecurity information on veterinarians and veterinary nurses.

Clinic protocols and veterinarians within the workplace were identified by participants as the most influential sources of biosecurity information for both veterinarian and veterinary nurse cohorts (Fig 3).

## Discussion

This study investigated the knowledge, attitudes and practices of Australia’s veterinary workforce regarding Q fever disease and vaccination and made comparisons between veterinarians and veterinary nurses. The key finding of this study was a shortfall in Q fever vaccine uptake among the veterinary nursing cohort, with less than a third of participants reporting they had sought vaccination. This was particularly disconcerting as the majority of veterinary nurses participating in the survey were women of child bearing age, who could potentially face an increased risk of chronic disease outcomes if they were to contract Q fever while pregnant [8]. In contrast, the majority of veterinarians had sought vaccination with uptake similar to that observed in abattoir workers during Australia’s National Q Fever Vaccination Program [49] and for other occupational diseases among medical professions [50]. The discrepancy between veterinarians and veterinary nurses seeking vaccination was observed despite the overwhelming majority of both cohorts agreeing that Q fever is a serious disease and that vaccinations are important for prevention of serious diseases. It is important to understand why this gap in vaccine uptake exists to improve Q fever vaccine uptake by veterinary nurses within Australia and to inform the potential introduction of the Q fever vaccine to at-risk cohorts internationally.

Some answers to the question of why veterinary nurses have a lower Q fever vaccination rate were evident from the study and can be supported by theories of health behaviour models [51]. The first step towards vaccination primarily requires some knowledge and awareness of both the health risk Q fever poses and the availability of a vaccine [51,52]. Veterinary nurses however reported a notable lack of awareness of the Q fever vaccine and a particularly low level of self-reported Q fever knowledge. Since veterinary nurses identified workplace protocols and veterinarians within their workplace as the two main sources from which they obtained biosecurity information, the shortfall in the two fundamental areas of awareness and knowledge may point to a failure of workplace health and safety (WH&S) protocols in the practices in which these veterinary nurses were employed as well as a failure of their veterinary colleagues to provide adequate information and WH&S training regarding Q fever. This is consistent with other studies calling for improved WH&S training within veterinary clinics both in Australia and internationally [39,53–57]. Once aware of the risks associated with Q fever and the availability of a Q fever vaccination, the formation of an intention to seek vaccination relies on an individual’s attitudes, both positive and negative, associated with seeking and receiving the vaccination. These attitudes would include one’s perceived susceptibility to Q fever disease, perceived severity of Q fever disease, perceived benefits of vaccination and perceived barriers to vaccination [51]. A lack of perceived susceptibility and severity was evident among non-vaccinates in both cohorts, with the belief that they personally were not at risk of being severely affected by Q fever the most influential reason for not seeking vaccination. This view may reflect a lack of knowledge of the sources of infection and therefore the potential level of exposure for veterinary workers. For example, some workers may not be aware that companion animals are a source of infection, believing that only livestock transmit the pathogen to humans. This is likely in the nursing cohort at least, given that a quarter of veterinary nurses surveyed identified that they did not know their potential risk of exposure to *C*. *burnetii* and close to one fifth, the majority of whom were engaged in companion animal work, stated nil exposure. Alternatively, those veterinary workers who might identify themselves as being at high risk of acquiring Q fever may assume that they have already been exposed to *C*. *burnetii* and are therefore immune and do not require vaccination.

Along with this lack of perceived vulnerability, perceived costs may be a key barrier to Q fever vaccination [58]. These costs may include those of time and financial expense; as well as difficulties involved in finding a Q fever vaccination provider as well as perceived and reported adverse outcomes following vaccination. The results of this study indicate all of these factors were somewhat influential barriers to vaccination for the participants of this study. These barriers however, were equally influential for both veterinarians and veterinary nurses. An understanding of the reasons behind the gap in vaccination between these two cohorts can be gained from the results of survey questions regarding reasons why participants sought vaccination.

The overwhelming majority of veterinarians in Australia received their Q fever vaccination as a compulsory requirement of their university studies. This usually occurs during the early years of veterinary or other animal science degrees, often with large numbers of students vaccinated on campus over a short time period or in organised vaccination clinics. Such vaccination programs reduce the cost of vaccination, the difficulty sourcing a trained provider and the time involved in being vaccinated. In addition, vaccination in this tertiary environment is driven by recommendation from peers and health and safety protocols, along with a desire to avoid both negative health and learning outcomes. Such adherence to subjective norms has been shown to be an integral component of health models, such as the theory of planned behaviour [59].

In contrast, veterinary nursing in Australia is typically taught in the workplace with only some nurses receiving supplementary or formal training via part time enrolment in technical tertiary institutions. As such, outside of the controlled learning environment experienced by veterinary students, veterinary nurses are typically not offered pre-arranged vaccination clinics at a reduced cost, which are proven to maximise vaccine uptake [60]. Instead, they are often required to seek out vaccination independently or rely on individual workplace vaccination programs which may or may not be available in their workplace. In the former group, the barriers to vaccination outlined above become much more influential, although there is anecdotal evidence that an increasing number of veterinary clinics are now approaching Q fever vaccination as their responsibility.

The Australian national Q fever vaccination program is further evidence of the success of vaccination *en mass*. The Australian government program funded the costs of screening and vaccination of more than 50,000 abattoir workers, sheep shearers, farm workers and their families. Workplace or community mass vaccination clinics were held, resulting in a program uptake ranging from 60 to 100% in the initial phase [49]. Such results suggest vaccination clinics targeting veterinary workers may help to improve vaccine uptake by veterinary nurses in Australia and initial vaccine uptake by veterinary workers if the vaccine were introduced internationally. However, the logistics of such a task would not be straight forward, as most veterinary clinics are small businesses as opposed to abattoirs and farming communities with a substantially larger workforce. Cooperation from many veterinary hospitals within a region would be required, highlighting the need for promotion and assistance from professional organisations, which in Australia could include the AVA or state veterinary registration boards.

This study also highlighted a potential issue surrounding lack of appropriate advice on Q fever and vaccination from general medical practitioners in Australia. In some instances it is reported that Q fever vaccination of apparently at risk individuals was actively discouraged. Further research is required to investigate the extent to which this is an issue among Australia’s general practitioners, beyond those reported in this survey, the results of which may have implications for vaccine introduction elsewhere.

Accessing the Australian veterinary workforce for the purpose of this study proved difficult due to the absence of a governing central body through which the workforce could be contacted. Despite state registration requirements for veterinarians, access via state veterinary boards was limited as was contacting individuals via the AVA. Veterinary nurses were only contactable by the Western Australia state veterinary board or the VNCA. It is expected that a greater number of responses may be achieved for both cohorts with uniform access to individuals, rather than businesses, as demonstrated by the national AVA workforce survey of veterinarians distributed annually by all state boards to individuals for online completion. The AVA workforce survey has achieved response rates varying from 15% to 29% of registered veterinarians. In comparison, this survey received responses from 8.4% of veterinarians registered in 2014, however it is not directly comparable since not all registered veterinarians were contactable in the current study due to the inability to access them via state boards as was the case for the AVA surveys. Other veterinary workforce studies in Australia have relied upon data collection from professional conferences; however these typically achieve smaller sample sizes and greater selection bias among participants [39,61]. A novel approach to data collection was required for this study to maximise the number of responses and reduce selection bias in order to improve sample representativeness. Although this resulted in a non-uniform approach across all states and the inability to accurately assess response rate, the numbers achieved were remarkable.

The sample of veterinarians was considered representative of the workforce of veterinarians in Australia as demonstrated by the fact that the number of veterinarian respondents by state reflected similar proportions to the proportion of veterinarians registered in each state (S2 Table)[62]. In addition, the distribution of age and sex were comparable to those reported in AVA national workforce surveys and Australian government statistics [47,62–64]. The proportion engaged in each practice type was also similar to those reported in the AVA national workforce surveys [62–64].

Currently there is little information available on the demographics of the Australian population of veterinary nurses beyond Australian government employment statistics. These statistics report 99% of Australian veterinary nurses are female which was reflected in the results of this survey [48]. However, the veterinary nurse respondents in this study were older and more highly educated than those reflected in government statistics and the states of Western Australia and New South Wales may be over-represented in this study while South Australia and Queensland appear to be under-represented (S2 Table) [48]. Coverage bias associated with web-based surveys is expected to be minimal due to the option of alternative methods for survey participation and the context of contact via the workplace and professional associations [65].

The results of this study most likely indicate a ‘best-case’ scenario for this workforce for a number of reasons. Firstly, a selection bias may have occurred towards participation from those familiar with the subject and/or with a higher level of concern about Q fever [66]. Secondly, the veterinary nursing participants in this study had a higher level of formal qualifications than their profession as a whole. As a result, the knowledge and awareness of Q fever could be expected to be lower in the greater population of veterinary workers, particularly veterinary nurses. Additionally, self-reported levels of knowledge tend to be over-claimed [67].

## Conclusion

Q fever vaccination uptake among veterinary nurses in Australia is well below uptake in veterinarians. The major barriers to vaccine uptake among the veterinary nurses participating in the study included a lack of knowledge and awareness of Q fever disease and availability of vaccination. Additionally, most veterinary nurses were either not aware of their risk of Q fever or reported nil perceived risk of exposure to *C*. *burnetii*. Veterinarians and clinic protocols were reported as the main source of biosecurity information to veterinary nurses, however the low levels of knowledge and uptake of the Q fever vaccine suggests that veterinarians and clinics were not providing adequate WH&S information and training, in relation to Q fever, as is required by Australian law. Other barriers to vaccination included financial expense, time, and difficulty in finding a Q fever vaccine provider; these were influential on veterinarians also. Veterinarians were mostly vaccinated in mass vaccination clinics during tertiary education, reducing the impact of these barriers, and indicating the potential for workplace vaccination clinics to improve the vaccination status of veterinary nurses. This study highlights the need for additional studies to identify the drivers behind seeking and recommending vaccination so that further recommendations on improving Q fever vaccine uptake by Australian veterinary workers can be made. Evaluation of the knowledge and attitudes towards this vaccine among the medical profession may also be warranted. Until then, some veterinary workers remain unnecessarily at risk and unaware of the dangers posed to them by *C*. *burnetii* while veterinarians and clinics may be failing to provide adequate duty of care.

## Supporting Information

S1 Table Education and veterinary work location reported by veterinary nurses and veterinarians surveyed in Australia in 2014. (DOC)

S2 Table Distribution of veterinarians and veterinary nurses by state; a comparison of respondents to available employment and registration data for Australia’s veterinary workforce in 2014. (DOC)
