# Supplementary material for: Q Fever Knowledge, Attitudes and Vaccination Status of Australia’s Veterinary Workforce in 2014
Source: PLoS One. 2016 Jan 12;11(1):e0146819. doi: 10.1371/journal.pone.0146819 (PMC4710533; doi:10.1371/journal.pone.0146819)
Supplement: S1 Table — (DOC) [file pone.0146819.s001.doc]

**Table S1. Education and veterinary work location reported by veterinary nurses and veterinarians surveyed in Australia in 2014**

|  | **Veterinarians (n = 890)** | **Veterinary Nurses (n=852)** |
| --- | --- | --- |
|  | **No.(%)*** | **No.(%)*** |
| **Highest level of education - veterinary nurses** | | |
| Certificate IV | n/a | 546 (64%) |
| Certificate II | n/a | 51 (6%) |
| Diploma/Bachelors/other | n/a | 132 (15%) |
| Nil | n/a | 109 (13%) |
| Not specified | n/a | 14 (2%) |
| **Highest level of education - veterinarians** | | |
| Undergraduate | 571 (65%) | n/a |
| Grad Certificate/Diploma | 111 (13%) | n/a |
| Masters | 50 (6%) | n/a |
| ANZCVS^a^ or equivalent | 95 (11%) | n/a |
| PhD or fellowship | 52 (6%) | n/a |
| Not specified | 11 (1%) | n/a |
| **University attended - veterinarians** | | |
| The University of Sydney | 223 (25%) | n/a |
| The University of Melbourne | 158 (18%) | n/a |
| Murdoch University | 150 (17%) | n/a |
| The University of Queensland | 207 (23%) | n/a |
| Charles Sturt University | 28 (3%) | n/a |
| James Cook University | 21 (2%) | n/a |
| The University of Adelaide | 4 (<1%) | n/a |
| Other (international) | 99 (11%) | n/a |
| **State of employment** |  |  |
| Queensland | 151 (17%) | 187 (22%) |
| New South Wales / Australian Capital Territory | 292 (33%) | 307 (36%) |
| Victoria | 201 (23%) | 163 (19%) |
| South Australia | 20 (2%) | 51 (6%) |
| Tasmania | 48 (5%) | 8 (<1%) |
| Western Australia | 156 (18%) | 114 (13%) |
| Northern Territory | 7 (<1%) | 5 (<1%) |
| Not specified | 15 (2%) | 17 (2%) |
