# Supplementary material for: Q Fever Knowledge, Attitudes and Vaccination Status of Australia’s Veterinary Workforce in 2014
Source: PLoS One. 2016 Jan 12;11(1):e0146819. doi: 10.1371/journal.pone.0146819 (PMC4710533; doi:10.1371/journal.pone.0146819)
Supplement: S2 Table — (DOC) [file pone.0146819.s002.doc]

**Table S2. Distribution of veterinarians and veterinary nurses by state; a comparison of respondents to available employment and registration data for Australia’s veterinary workforce in 2014.**

|  | **Veterinarians** | | | **Veterinary Nurses** | |
| --- | --- | --- | --- | --- | --- |
|  | **Study respondents (n = 890)** | **Veterinarians registered by state 2014^a^** | **ABS data 2014** | **Respondents (n=852)** | **ABS data 2014** |
| **State** | n (%) | n (%) | % | n (%) | % |
| Queensland | 151 (17%) | 2503 (24%) | 18.1 | 187 (22%) | 27.3 |
| New South Wales / Australian Capital Territory | 292 (33%) | 3203 (30%) | 42.1 | 307 (36%) | 17.2 |
| Victoria | 201 (23%) | 2586 (24%) | 24.7 | 163 (19%) | 22.9 |
| South Australia | 20 (2%) | 655 (6%) | 7.2 | 51 (6%) | 15.5 |
| Tasmania | 48 (5%) | 252 (2%) | 2.6 | 8 (<1%) | 5.6 |
| Western Australia | 156 (18%) | 1296 (12%) | 4.6 | 114 (13%) | 9.3 |
| Northern Territory | 7 (<1%) | 134 (1%) | 0.6 | 5 (<1%) | 1 |
| Not specified | 15 (2%) | **-** | - | 17 (2%) | - |

^a^Registration data reported within the Australian Veterinary Association veterinary workforce survey (2014). ABS; Australian Bureau of Statistics (2014).
